# Clinical Decision Support System to Enhance Quality Control of Spirometry Using Information and Communication Technologies

**DOI:** 10.2196/medinform.3179

**Published:** 2014-10-21

**Authors:** Felip Burgos, Umberto Melia, Montserrat Vallverdú, Filip Velickovski, Magí Lluch-Ariet, Pere Caminal, Josep Roca

**Affiliations:** ^1^Hospital Clinic - IDIBAPS - CiberesRespiratory Diagnostic CenterUniversity of BarcelonaBarcelonaSpain; ^2^Centre de Recerca en Enginyeria Biomèdica (CREB-UPC)Universitat Politècnica de CatalunyaBarcelonaSpain; ^3^Barcelona Digital Technology CentreBarcelonaSpain; ^4^ViCOROBUniversitat de GironaGironaSpain; ^5^Departament d'Enginyeria Telemàtica (ENTEL)Universitat Politècnica de CatalunyaBarcelonaSpain

**Keywords:** spirometry, telemedicine, information communication technologies, primary care, quality control

## Abstract

**Background:**

We recently demonstrated that quality of spirometry in primary care could markedly improve with remote offline support from specialized professionals. It is hypothesized that implementation of automatic online assessment of quality of spirometry using information and communication technologies may significantly enhance the potential for extensive deployment of a high quality spirometry program in integrated care settings.

**Objective:**

The objective of the study was to elaborate and validate a Clinical Decision Support System (CDSS) for automatic online quality assessment of spirometry.

**Methods:**

The CDSS was done through a three step process including: (1) identification of optimal sampling frequency; (2) iterations to build-up an initial version using the 24 standard spirometry curves recommended by the American Thoracic Society; and (3) iterations to refine the CDSS using 270 curves from 90 patients. In each of these steps the results were checked against one expert. Finally, 778 spirometry curves from 291 patients were analyzed for validation purposes.

**Results:**

The CDSS generated appropriate online classification and certification in 685/778 (88.1%) of spirometry testing, with 96% sensitivity and 95% specificity.

**Conclusions:**

Consequently, only 93/778 (11.9%) of spirometry testing required offline remote classification by an expert, indicating a potential positive role of the CDSS in the deployment of a high quality spirometry program in an integrated care setting.

## Introduction

### High Quality Spirometry Testing

High quality spirometry testing across health care levels is pivotal for proper management of patients with prevalent chronic respiratory disorders, namely asthma and chronic obstructive pulmonary disease (COPD) [1].

We have recently reported the effectiveness of a Web-based application for remote offline expert support to enhance the quality of spirometry in primary care. High quality testing improved in a sustainable manner with the remote support [2]. A relevant difference was observed between the intervention group, 2419/3383 (71.50%) high quality spirometry, and the control group, 713/1198 (59.52%) high quality spirometry, throughout the 12 month follow-up period (*P*<.001). Similar figures have been obtained in pharmacy offices, as part of a COPD case finding program [3].

In the Basque Country (Spain), the ongoing regional deployment of the Web-based offline support program from specialists to primary care will cover the entire population, 2.2 million inhabitants, by the end of 2014 [4,5]. Interestingly, their results [6] are similar to those reported in the initial randomized controlled trial [2] described above.

Ideally, extensive deployment of a high quality spirometry program in the community should offer accessibility to standardized raw spirometric data through a technological architecture providing interoperability across health care tiers. To this end, a Clinical Document Architecture for spirometry using Health Level Seven v3 standards was recently made available by the Catalan Health Department [7], such that spirometric testing will be available at the regional level. In this scenario, automatic assessment of quality of spirometry testing should enhance the efficiency of the program. Unfortunately, current applications for online assessment of quality of spirometry misclassify the tests, as compared with examinations done by expert professionals [2].

### Clinical Decision Support System

We hypothesize that elaboration and validation of a clinical decision support system (CDSS) for online automatic assessment and certification of quality of spirometry in primary care may represent a pivotal step toward regional adoption of the high quality spirometry program with an integrated care approach.

The current study is part of the refinement of the ongoing deployment of the high quality spirometry program in Catalonia [8], an European region of 7.5 million inhabitants.

## Methods

### Building-Up the Clinical Decision Support System

**Figure 1:**
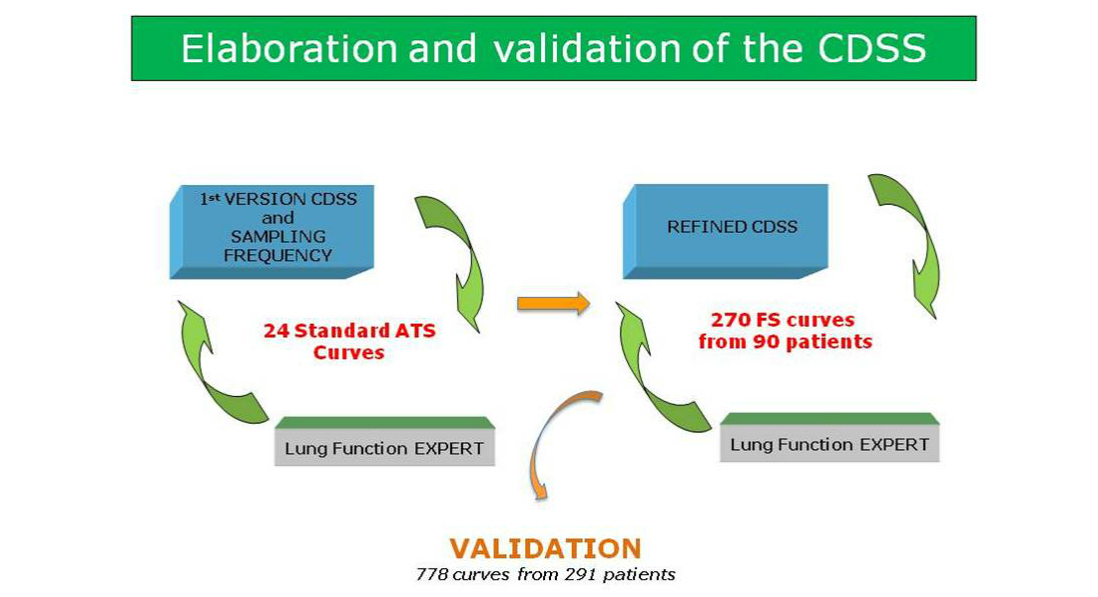
Flow of the process followed to elaborate and validate the Clinical Decision Support System (CDSS). ATS=American Thoracic Society; FS=forced spirometry.

Figure 1 shows the initial step in the process for elaboration of the CDSS was the identification of the optimal sampling frequency to achieve the highest sensitivity and specificity in the analysis of the spirometric curves. To this end, a systematic examination of a large range of sampling frequencies, from 6.25 Hz to 100 Hz, was done during the first iterative process.

The process was done using the 24 standard flow-volume and volume-time curves from the pulmonary waveform generator recommended by the American Thoracic Society/European Respiratory Society (ATS/ERS) [7]. This set of 24 standard curves cover the entire spectrum of clinical abnormalities, as well as common spirometric artifacts. They are used as a reference material for calibration purposes and, in general, to facilitate comparisons among lung function laboratories.

The construction of an initial version of the CDSS was carried out using the 24 standard spirometry curves [9,10] following an iterative process, as displayed in Figure 1. In each step, the results generated by the CDSS were compared with the criteria of one expert in the field of lung function testing (FB), and the iterative process was maintained until sensitivity and specificity of the results generated by the CDSS showed 24/24 (100.0%) agreement with the expert.

The CDSS combines the different aspects assessed on the spirometry curve in one score with three different categories: (1) grade 0, rejected due to unacceptable morphology of the spirometry curve; (2) grade 1, acceptable for further classification according to Table 1; or (3) grade 2, undefined characteristics of the spirometry (see Multimedia Appendix 1 for examples of the three categories in Figure 1S). The two first categories, grades 0 and 1, allow proper online automatic classification of spirometry testing as well as the generation of a certified spirometry curve to be potentially shared across health care tiers; whereas grade 2 requires offline expert assessment.

**Table 1:** Quality scores for spirometric maneuvers according to ATS/ERS standardization [9].

Scores	Maneuvers
A^a^	3 acceptable maneuvers, and best 2 matched with differences in FVC^b^and/or FEV_1_<150 ml
B	3 acceptable maneuvers, and best 2 matched with differences in FVC^b^and/or FEV_1_^c^<200 ml
C	2 acceptable maneuvers, and best 2 matched with differences in FVC and/or FEV_1_^c^<250 ml
D	1 acceptable maneuver
F	No acceptable maneuvers

^a^High quality spirometries, A and B scores, correspond to A, 3 acceptable maneuvers with differences in FVC and/or FEV_1_<150 ml; and B, 3 acceptable maneuvers with differences in FVC and/or FEV_1_<200 ml; C, to high variability among maneuvers; D, only one acceptable maneuver; and F no acceptable maneuver.

^b^FVC = forced vital capacity

^c^FEV_1_= forced expiratory volume in the first second

### The Characteristics and the Algorithm

The CDSS systematically assessed 27 different characteristics of each spirometry curve, as displayed in Table 2. There were four out of the 27 characteristics that were extracted from the international recommendations for standardization of the test, jointly reported by the ATS and the ERS [11]; whereas the remaining 23 were introduced during the current research. Each of these 27 features had a well defined specific algorithm for calculations. The mathematical description of a feature constituted the so-called metric. It is of note that a given feature may require more than one metric. The quantitative values of a given metric were denominated thresholds that were used for quality assessment. It is also of note that some metrics may have primary and secondary thresholds. The initial parameters of the automatic algorithm for online assessment of quality of spirometry were refined through successive iterations until the final version of the CDSS was obtained (Figure 1). As indicated above, the performance of each of the successive versions of the CDSS was compared with the results provided by the expert. A refined version of the CDSS was achieved using 270 curves from 90 patients from [2].

**Table 2:** List of criteria of the forced spirometry curve explored by the CDSS.

Forced spirometry curve	Criteria^i^
BEV^a^ trad	Back extrapolation >0.15 L or < 5% of FVC^g^
EOTV^b^ trad	End of test criteria, volume < 0.025 L in time ≥1 s
Tex^c^	Time of end FVC^g^(Tex>6 s)
EOTV^b^ new (5 criteria)	a) EOTV^b^< 0.025 L or Tex^c^>6 s; b) If Tex^c^>6 s EOTV^b^<0.025 L in time 0.5 s; c) If Tex^c^>6 s, EOTV^b^< 0.1 L; d) EOTV^b^(Tex^c^) < 0.025 L; and e) EOTV^b^< 0.025 * Tex/6 L
Peak_Valley_Single	High local maximum (peak) and minimum (valley) in FV^e^curve
Peak_Valley_Combined	High local maximum (peak) and minimum (valley) in FV^e^curve close to FEV_1_^h^
VT^d^ end	Irregularity or oscillation at the end of FT^m^curve
FV^e^_slope_single	Variation of FV^e^slope or high FV^e^slope
FV^e^_slope_combined	Variation of FV^e^slope and high FV^e^slope
FV^e^Slope_Test_Combo	Irregularity and variation of FV^e^slope or high FV^e^slope
FV^e^Slope_Test_Combo_Area Under Rect^j^	Irregularity or variation of FV^e^slope and high FV^e^slope
FV^e^Slope_Test_Combo4	Irregularity and variation of FV^e^slope and high FV^e^slope
Diff_single^k^	Irregular concavity-convexity before the PEF^f^value in FV^e^curve
Diff_combined^l^	Irregular slope and irregular concavity-convexity before the PEF^f^value in FV^e^curve
PEF^f^ TimeUp	Time to archive PEF^f^< 130 milliseconds
PEF^f^ TimeDown	Time to archive PEF^f^> 0.25 milliseconds
PEF^f^ Cut	PEF^f^is not a peak in FV^e^curve (is plane), volume (F^n^=PEF^f^) > 15 % FVC^g^
PEF^f^ Cut2 FEV_1_^h^	PEF^f^is not a peak in FV^e^curve (is plane), volume (F^n^=PEF^f^) > 17.5 % FEV_1_^h^
PEF^f^ DoublePeak	PEF^f^bimodal in FV^e^curve
PEF^f^ Slow	Volume to archive PEF^f^< 20% FVC^g^

^a^BEV = back extrapolation

^b^EOTV = end of test criteria, volume

^c^Tex = Time to end FVC

^d^VT = volume/time curve

^e^FV = flow/volume curve

^f^PEF = peak expiratory flow

^g^FVC = forced vital capacity

^h^FEV_1_= forced expiratory volume in the first second

^i^The list includes the classical parameters used by ATS/ERS guidelines [11].

^j^Rect = rectum

^k^Diff single= irregular concavity-convexity before the PEFf value in flow volumen curve concavity or convexity exists if the extracted signal metric

^l^Diff_combined = irregular slope and irregular concavity-convexity before the peak expiratory flow value in flow volume curve

^m^FT = flow/time curve

^n^F=flow

### Clinical Decision Support System Validation

The refined version of the CDSS was compared with a database of 778 curves from 291 patients from one of the primary care centers in Barcelona. The spirometry testing was done using a spirometer (Sibel 120, SIBELMED, Barcelona Spain). Again, the score generated by the CDSS was compared with the one obtained from the same expert evaluator.

The use of the two patient databases, for refinement and validation purposes, was approved by the Ethical Committee of the Hospital Clínic i Provincial de Barcelona.

### Data Analysis

**Figure 2:**
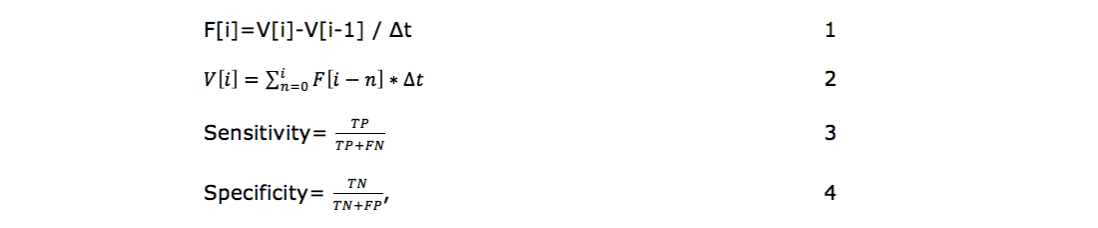
Equations for data analysis. F=flow; V=volume; i=1,…,N; N=length of the sequence; true positive (TP) corresponds to curves classified as grade 0 by both CDSS and the evaluator; true negative (TN) corresponds to curves classified as grade 1 by the CDSS and the by the evaluator; false positive (FP) indicates curves classified as grade 0 by the CDSS, but classified in grade 1 by the evaluator; and, false negative (FN) corresponds to curves classified as grade 1 by the CDSS, but as grade 0 by the evaluator.

The ATS database [10] contains volume (*V*) values of each curve, from which flow (*F*) values were obtained by discrete differentiation (equation 1, Figure 2). The two patient’s databases contained *F* values, from which *V* values were obtained by discrete integration (equation 2, Figure 2). The sample period is Δt=0.01s, so the sample frequency is 100 Hz. Sensitivity and specificity of the CDSS were calculated for all curves classified as grades 0 or 1 using equations 3 and 4 in Figure 2.

## Results

### The Sampling Frequency

The sampling frequency that provided the highest sensitivity and specificity for the analysis carried out with the 24 standard spirometry curves recommended by the ATS [10] was 100 Hz (Figure 1 and Multimedia Appendix 2, Table 2S), this frequency is widely used in commercial spirometers, and it is reasonable from the electronic transferability point of view. This result was confirmed in the 270 curves from 90 subjects [2].

Both sensitivity and specificity of the CDSS were initially calculated with the 24 standard spirometry curves recommended by the ATS [11] using only grade 0 and grade 1 curves. The results were as follows, grade 0, n=15; grade 1, n=6; grade 2, n=3 with 24/24 (100.0%) sensitivity and 24/24 (100.0%) specificity. Up to five complete versions of the CDSS were generated in the two iterative processes indicated in Figure 1, until a final version of the CDSS was ready for validation.

### Grading the Curves

The validation study using 778 curves from 291 patients showed the following distribution of spirometry curves, 419/778 maneuvers (53.8%) were appropriately classified as bad curves (grade 0); 266/778 maneuvers (34.2%) were appropriately classified as good curves (grade 1); and only 93/778 maneuvers (11.9%) needed an offline review by a lung function expert to assess quality of the test (grade 2; see Multimedia Appendix 3). Sensitivity and specificity calculations for grade 0 and grade 1 curves were 96.1 and 94.9%, respectively.

## Discussion

### The Current Research

The current research has generated and validated a CDSS that shows the ability to classify a reasonable percentage of spirometry curves, 685/778 (88.1%) as either acceptable (grade 1) or bad maneuvers (grade 0). Only 93/778 (11.9%) of the curves were classified as undefined (grade 2) and were candidates for offline remote validation by an expert. Moreover, we observed that both sensitivity and specificity of the CDSS were very high. Consequently, the results seem to indicate that a vast majority of spirometry testing carried out by nonspecialized professionals in primary care can be reliably assessed online, and the high quality spirometry program partly based on remote automatic evaluation of the testing could be considered ready for regional scalability. Obviously, further steps toward extensive deployment of the program must be planned with caution. A proper monitoring of the potential for generalization of the current results and the need for further refinements of the current CDSS should be taken into account.

The results of the current research overcome some of the limitations of the existing computer-based algorithms generating automatic feedback, as reported in [2,12]. It is acknowledged, however, that automatic feedback based on enhanced algorithms like the one proposed by the current research may be effective only if they are part of a comprehensive program for high quality forced spirometry.

In the new scenario, as indicated by the business process management notation (BPMN) diagram (Multimedia Appendix 2, Figure 2S), acceptable maneuvers (grade 1) will be automatically addressed to the algorithm indicated in Table 1 that classifies and certifies spirometry testing prior to its recording into the local (electronic health record) and regional repositories. In contrast, those maneuvers classified as bad curves (grade 0) will generate an online specific error message to the professional, indicating the need to perform additional testing until quality acceptance is reached. As indicated, we estimate that approximately 12% of the curves will not be properly classified (grade 2), and they will need an offline remote supervision by an expert professional. In this case, the spirometry testing of a given patient may need to be rescheduled.

Previous reports have indicated the potential of telemedicine to enhance both quality and diagnostic potential of spirometry testing carried out by nonexpert professionals [13-15], but the quality control in those studies was based on offline analyses by expert professionals carried out in a time consuming manner [16-18]. Likewise, the need for an external, likely centralized, quality control program [15,17-20] is well established. The results of the current study refine previous achievements [2] and open the way to explore extensive and efficient adoption of this type of high quality spirometry programs.

We acknowledge that high quality spirometry programs combine several different dimensions, namely: (1) professional coaching [21,22]; (2) remote support [2]; (3) interoperability of testing across health care levels [20]; (4) standards for procurement of equipment [11,23]; and (5) support to interpretation of testing [24,25]. The current study provides pivotal results to efficiently address issues associated to remote support of spirometry testing. But, a proper integration of all the above elements needs to be considered in the process of shaping a successful high quality spirometry program for scalability at regional level.

### Limitations of the Study

We acknowledge two principal limitations of the study. First, we included only one expert observed (FB). The CDSS should be reassessed in the future with the inclusion of at least 3 different experts. Moreover, the current study evaluates the CDSS in an isolated manner. But, further assessment of the whole clinical process as defined in the BPMN (see Multimedia Appendix 2, Figure 2S) should be done before specific plans for scalability are undertaken.

### Conclusions

To our knowledge, the current study constitutes the first successful attempt to validate an automatic CDSS for large scale online assessment of quality of spirometry testing. The incorporation of the CDSS into the Web-based application for remote assistance to primary care professionals [2] may facilitate sustainable high quality spirometry generating a significant added value in an integrated care scenario.

The results indicate a high potential of the CDSS for discrimination between good and poor quality results of spirometry testing, but they require further independent validation before specific plans for implementation are materialized.

## Multimedia Appendix 1

The algorithm for computing maneuver acceptability, using the 27 set of criteria.

## Multimedia Appendix 2

Three examples with curves classified as Grade 0, 1 and 2. The Business Process Model Notation (BPMN) diagram displays the use of the CDSS for quality control in primary care within a coordinated care scenario. The results of the protocol undertaken to identify the optimal sampling frequency during the first iterative process are shown here.

## Multimedia Appendix 3

For each FS curve, the results generated by the CDSS are compared with those provided by the expert professional. It is of note, that only the expiratory portion of the FS manouevres was taken into account for analysis.

## Abbreviations

ATS: American Thoracic Society
BPMN: business process management notation
CDSS: clinical decision support system
COPD: chronic obstructive pulmonary disease
ERS: European Respiratory Society
