# Supplementary material for: Clinical Decision Support System to Enhance Quality Control of Spirometry Using Information and Communication Technologies
Source: JMIR Med Inform. 2014 Oct 21;2(2):e29. doi: 10.2196/medinform.3179 (PMC4288080; doi:10.2196/medinform.3179)
Supplement: Supplementary file 1 [file medinform_v2i2e29_app1.pdf]

Multimedia Appendix 1

Table 1S describes the algorithm for computing maneuver acceptability, using the 27 set of criteria indicated in **Table 2**

Compute Maneuver Acceptability

Inputs

```
flow-time        # Spirometry Curve
Criteria[1..27]  # 27 sets of criteria

volume-time ← f_1(flow-time)
flow-volume ← f_2(flow-time)
AcceptabilityCategory ← GRADE 1
i ← 1
while (AcceptabilityCategory ≠ GRADE 0) AND (i ≤ 27)
  Parameters_i ← f_3(Criteria_i, flow-time, volume-time, flow-volume)
  ValidationResult ← Validate(Criteria_i, Parameters_i)
  if ValidationResult = REJECT
    AcceptabilityCategory ← GRADE 0
  else if ValidationResult = UNSURE
    AcceptabilityCategory ← GRADE 2
  end if
end if
i ← i+1
end while
return AcceptabilityCategory
```

f_1 converts a flow-time curve into a volume-time curve

f_2 converts a flow-time curve into a flow-volume curve

f_3 extracts a set of parameters for a given criteria set from the flow-time, volume-time, flow-volume curves

Validate checks a given parameter set against a given criteria set, returns *REJECT* when the parameter set DO NOT satisfy the given criteria set and the maneuver should be rejected outright irrespective of the other criteria sets; returns *UNSURE* when the parameter set cannot automatically be validated against the given criteria set thus the maneuver should be reviewed by an expert; returns ACCEPTABLE when the given parameter set satisfies the given criteria set.
