# Supplementary material for: Clinical Decision Support System to Enhance Quality Control of Spirometry Using Information and Communication Technologies
Source: JMIR Med Inform. 2014 Oct 21;2(2):e29. doi: 10.2196/medinform.3179 (PMC4288080; doi:10.2196/medinform.3179)
Supplement: Supplementary file 2 [file medinform_v2i2e29_app2.pdf]

Multimedia Appendix 2

Figure 1S. Examples of grades: 0 (A), 1(B) and 2 (C)

(A)

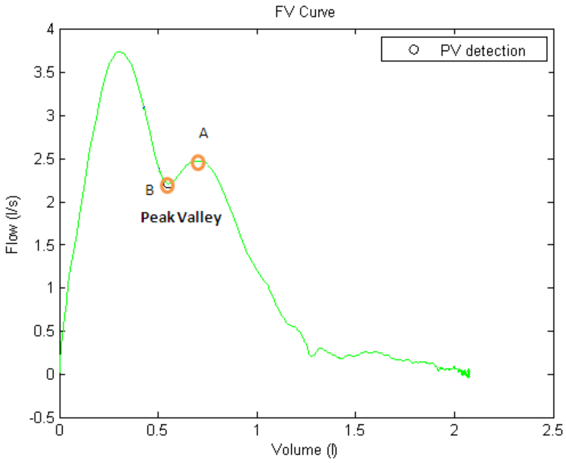

Peak Valley = F_A-F_B

Figure 1S (A) classified as grade 0, bad quality, due to fluctuations of the expiratory flow

(B)

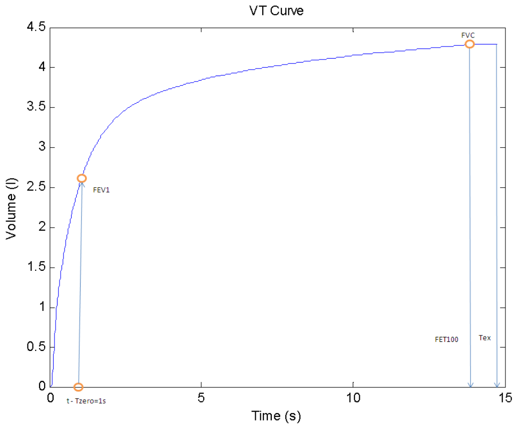

FVC, FEV1, FET100 and Tex

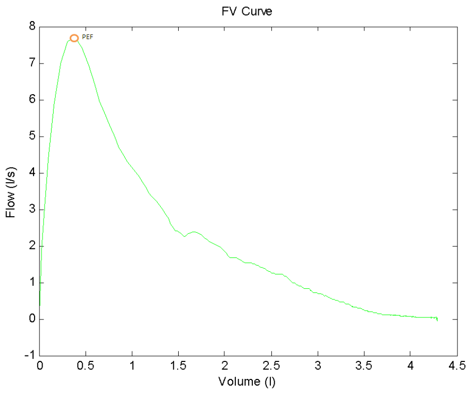

PEF

Figure 1S (B) classified as grade 1, good quality, it shows a smooth decrease of flow after the peak expiratory flow.

(C)

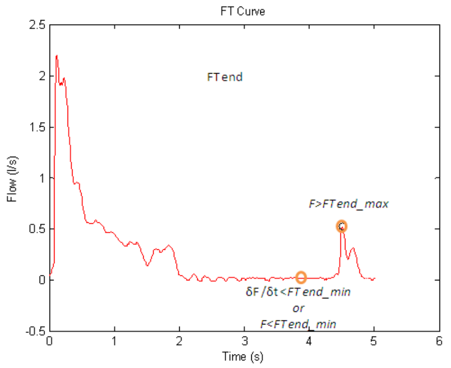

FT end

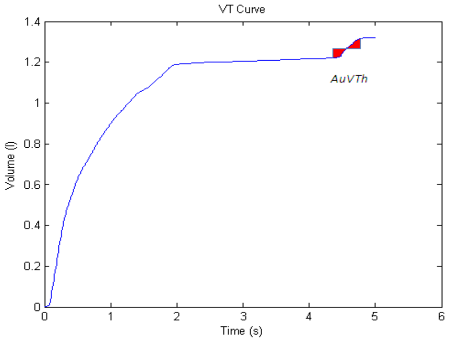

Area between horizontal line and Volume Curve in the point detected by *FT end*

Figure 1S (C) classified as grade 2, quality uncertain, fluctuations at end of the test zone generates uncertainty about quality of the spirometry.

Table 2S Sensitivity and specificity results obtained at different sampling frequencies.

	Sample Frequency (Hz)	Sen (%)	Spe (%)	Number of Curves in Grade 2
Analysis 1 Frequency FV Curve of 100 ℓ^{-1}	100	96.1	94.9	93
	50	94.4	93.1	95
	25	91.9	91.3	95
	12.5	89.1	83.9	180
	6.25	96.3	33.9	388
Analysis 2 Frequency FV Curve equal to Frequency VT Curve	100	96.1	94.9	93
	50	91.8	95.4	98
	25	84.5	78.4	174
	12.5	92.4	27.1	141
	6.25	100	1.98	108
Analysis 3 Frequency FV Curve of 200 ℓ^{-1}	100	91.7	88.6	87
	50	89.8	96.1	104
	25	86.2	94.0	98
	12.5	82.9	89.2	184
	6.25	96.4	31.0	371

Sen, sensitivity; Spe, specificity. The highest sensitivity and specificity were observed with a sampling frequency of 100 Hz for VT and FT curves, and 100 ℓ-1 for the FV curve. Grade 2 need an off-line review.

Figure 2S – Spirometry transfer to Primary Care. The figure depicts the clinical process of a patient with respiratory symptoms attending a primary care visit. The flow is as follows: the general practitioner (GP) decides that the patient is a candidate for

spirometry testing and both baseline and post-bronchodilator studies will be done by a non-specialized nurse. At the end of the testing, the results will be automatically assessed using the algorithm that will generate spirometry certification for quality. The three possible outcomes are: i) the spirometry testing qualifies as high quality. It will be used by the GP for his/her decision-making process and the certified spirometry will be sent to the patient's electronic health record (EHR) and to the regional repository (shared EHR or HC3, Historia Clinica Compartida de Catalunya); ii) the FS testing does not fulfill quality criteria. Then automatic feedback with specific information on the problem is forwarded to the nurse while the patient is still on site. Consequently, the nurse will have the opportunity to solve the problem and generate a high-quality spirometry test; and, iii) approximately 12% of the spirometry testing will be classified as undefined by the automatic algorithm and forwarded to the specialist for advice. The specialist will provide remote off-line recommendations directly to both the GP/nurse and the patient will be attended by the GP who will take the final decision on how to proceed. In two of these scenarios, the first and the third, the certified spirometry will be forwarded to the regional EHR (or HC3).

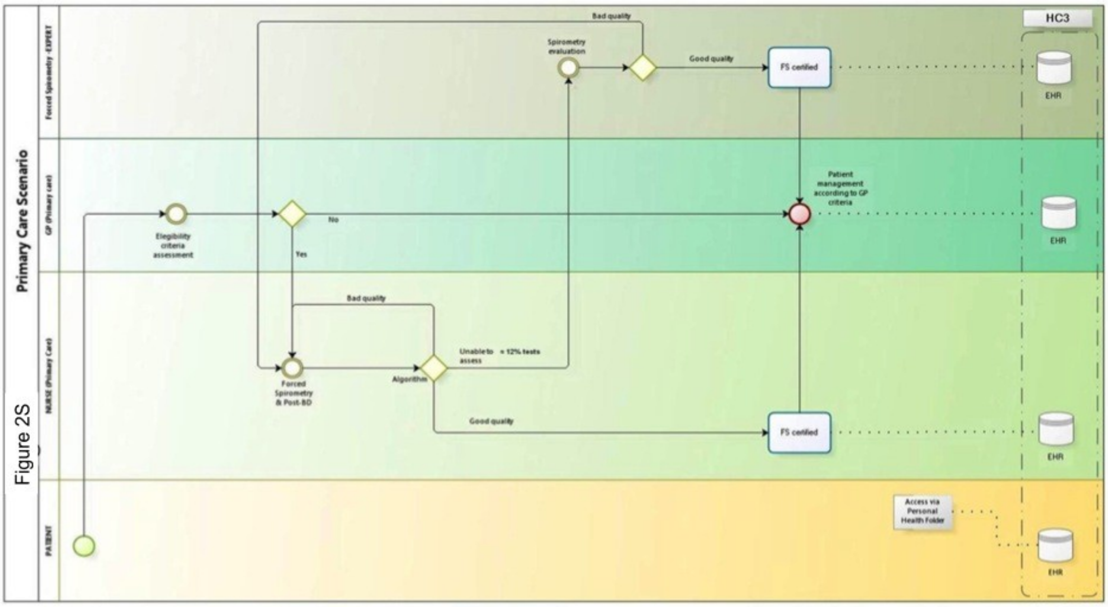

Figure 2S
