# Supplementary material for: Clinical Decision Support System to Enhance Quality Control of Spirometry Using Information and Communication Technologies
Source: JMIR Med Inform. 2014 Oct 21;2(2):e29. doi: 10.2196/medinform.3179 (PMC4288080; doi:10.2196/medinform.3179)
Supplement: Supplementary file 3 [file medinform_v2i2e29_app3.pdf]

Multimedia Appendix 3

Table 3S compares, for each FS curve, the results generated by the CDSS with those provided by the expert professional. It is of note, that only the expiratory portion of the FS manouevres was taken into account for analysis

N CURVE	ID PATIENT	N TRIAL	CDSS	EXPERT
1	'1013786A '	'M1'	2	1
2	'1013786A '	'M2'	0	0
3	'1013786A '	'M3'	2	1
4	'1030941A '	'M1'	1	1
5	'1030941A '	'M2'	1	1
6	'1030941A '	'M3'	1	1
7	'1037227A '	'M1'	1	1
8	'1037227A '	'M2'	1	1
9	'1037227A '	'M3'	1	1
10	'1046835A '	'M1'	1	1
11	'1046835A '	'M2'	2	1
12	'1046835A '	'M3'	2	1
13	'1053154A '	'M1'	1	1
14	'1053154A '	'M2'	0	1
15	'1053154A '	'M3'	1	1
16	'1053154B '	' '	1	1
17	'1083852A '	'M1'	1	1
18	'1083852A '	'M2'	1	1
19	'1083852A '	'M3'	1	0
20	'1099918A '	'M1'	1	1
21	'1099918A '	'M2'	1	1
22	'1099918A '	'M3'	1	1
23	'119731A '	'M1'	1	1
24	'119731A '	'M2'	0	0
25	'119731A '	'M3'	0	0
26	'119731B '	' '	1	1
27	'1200930A '	'M1'	2	1
28	'1200930A '	'M2'	1	1
29	'1200930A '	'M3'	1	1
30	'1207147A '	'M1'	1	1
31	'1207147A '	'M2'	1	1
32	'1207147A '	'M3'	1	1
33	'1217829A '	'M1'	1	1
34	'1217829A '	'M2'	1	1
35	'1217829A '	'M3'	1	1
36	'1219563A '	'M1'	1	1
37	'1219563A '	'M2'	1	1
38	'1219563A '	'M3'	1	1
39	'1221269A '	'M1'	2	0
40	'1221269A '	'M2'	0	0
41	'1221269A '	'M3'	0	0
42	'1221269B '	' '	2	0
43	'1223722A '	'M1'	1	1
44	'1223722A '	'M2'	1	1
45	'1223722A '	'M3'	0	0
46	'1223722B '	'M1'	1	1
47	'1223722B '	'M2'	1	1
48	'1223722B '	'M3'	0	0
49	'1239178A '	'M1'	2	0
50	'1239178A '	'M2'	1	1
51	'1239178A '	'M3'	0	0
52	'1239178B '	' '	1	1
53	'1255183A '	'M1'	1	1
54	'1255183A '	'M2'	1	1
55	'1255183A '	'M3'	1	1
56	'1262625A '	'M1'	1	1

57	'1262625A	'	'M2'	0	0
58	'1262625A	'	'M3'	0	0
59	'1262625B	'	' '	0	0
60	'1265069A	'	'M1'	1	1
61	'1265069A	'	'M2'	2	0
62	'1265069A	'	'M3'	2	0
63	'1265069B	'	' '	1	1
64	'1278069A	'	'M1'	1	1
65	'1278069A	'	'M2'	1	1
66	'1278069A	'	'M3'	1	1
67	'1311538A	'	'M1'	0	0
68	'1311538A	'	'M2'	2	1
69	'1311538A	'	'M3'	0	0
70	'1311538B	'	'M1'	2	1
71	'1311538B	'	'M2'	0	0
72	'1311538B	'	'M3'	0	0
73	'1311538C	'	' '	1	1
74	'1352549A	'	'M1'	1	1
75	'1352549A	'	'M2'	1	1
76	'1352549A	'	'M3'	0	0
77	'1352549B	'	'M1'	2	1
78	'1352549B	'	'M2'	0	0
79	'1352549B	'	'M3'	0	0
80	'1352549C	'	' '	0	0
81	'1363389A	'	'M1'	0	1
82	'1363389A	'	'M2'	0	0
83	'1363389A	'	'M3'	0	0
84	'1363389B	'	'M1'	0	0
85	'1363389B	'	'M2'	2	0
86	'1363389B	'	'M3'	0	0
87	'1363389C	'	'M1'	0	0
88	'1363389C	'	'M2'	0	0
89	'1363389C	'	'M3'	0	0
90	'1365121A	'	'M1'	1	1
91	'1365121A	'	'M2'	1	1
92	'1365121A	'	'M3'	1	1
93	'1366379A	'	'M1'	1	1
94	'1366379A	'	'M2'	1	1
95	'1366379A	'	'M3'	1	1
96	'1370782A	'	'M1'	1	1
97	'1370782A	'	'M2'	1	1
98	'1370782A	'	'M3'	0	0
99	'1370782B	'	'M1'	1	1
100	'1370782B	'	'M2'	0	0
101	'1370782B	'	'M3'	1	1
102	'1370782C	'	' '	0	0
103	'1380347A	'	'M1'	1	1
104	'1380347A	'	'M2'	2	0
105	'1380347A	'	'M3'	0	0
106	'1380347B	'	'M1'	2	0
107	'1380347B	'	'M2'	0	0
108	'1380347B	'	'M3'	0	0
109	'1381326A	'	'M1'	2	1
110	'1381326A	'	'M2'	1	1
111	'1381326A	'	'M3'	2	1
112	'1381729A	'	'M1'	1	1
113	'1381729A	'	'M2'	1	1
114	'1381729A	'	'M3'	1	1
115	'1381729B	'	' '	0	0
116	'1383202A	'	'M1'	1	1
117	'1383202A	'	'M2'	1	1
118	'1383202A	'	'M3'	1	1
119	'1391515A	'	'M1'	2	1
120	'1391515A	'	'M2'	2	1
121	'1391515A	'	'M3'	0	0
122	'1391515B	'	'M1'	0	0
123	'1391515B	'	'M2'	0	0
124	'1391515B	'	'M3'	0	0
125	'1391515C	'	'M1'	0	0
126	'1391515C	'	'M2'	0	0

127	'1391515C '	'M3'	0	0
128	'1395202A '	'M1'	1	1
129	'1395202A '	'M2'	0	0
130	'1395202A '	'M3'	0	0
131	'139828A '	'M1'	1	1
132	'139828A '	'M2'	1	1
133	'139828A '	'M3'	1	1
134	'1400340A '	'M1'	1	1
135	'1400340A '	'M2'	1	1
136	'1400340A '	'M3'	1	1
137	'1404212A '	'M1'	0	0
138	'1404212A '	'M2'	0	0
139	'1404212A '	'M3'	0	0
140	'1404212A '	'M1'	0	0
141	'1404212A '	'M2'	0	0
142	'1404212A '	'M3'	0	0
143	'1404212B '	'M1'	1	1
144	'1404212B '	'M2'	1	1
145	'1404212B '	'M3'	0	0
146	'1404212C '	' '	1	1
147	'1405723A '	'M1'	2	0
148	'1405723A '	'M2'	1	1
149	'1405723A '	'M3'	2	1
150	'1410712A '	'M1'	2	0
151	'1410712A '	'M2'	1	1
152	'1410712A '	'M3'	1	1
153	'1412415A '	'M1'	1	1
154	'1412415A '	'M2'	1	1
155	'1412415A '	'M3'	0	0
156	'1412415B '	' '	1	1
157	'1416766A '	'M1'	1	1
158	'1416766A '	'M2'	2	1
159	'1416766A '	'M3'	1	1
160	'1418105AA '	'M1'	1	1
161	'1418105AA '	'M2'	1	1
162	'1418105AA '	'M3'	1	1
163	'1418166A '	'M1'	0	0
164	'1418166A '	'M2'	0	0
165	'1418166A '	'M3'	0	0
166	'1418166B '	'M1'	0	0
167	'1418166B '	'M2'	0	0
168	'1418166B '	'M3'	0	0
169	'1418166C '	' '	0	0
170	'1420181A '	'M1'	1	1
171	'1420181A '	'M2'	1	1
172	'1420181A '	'M3'	1	1
173	'1420340A '	'M1'	1	1
174	'1420340A '	'M2'	0	0
175	'1420340A '	'M3'	0	0
176	'1420340B '	' '	0	0
177	'1421120A '	'M1'	0	0
178	'1421120A '	'M2'	0	0
179	'1421120A '	'M3'	0	0
180	'1421120B '	'M1'	0	0
181	'1421120B '	'M2'	0	0
182	'1421120B '	'M3'	0	0
183	'1421120C '	' '	0	0
184	'142125A '	'M1'	0	0
185	'142125A '	'M2'	2	1
186	'142125A '	'M3'	0	0
187	'142125B '	'M1'	2	1
188	'142125B '	'M2'	0	1
189	'142125B '	'M3'	0	0
190	'142125B '	'M1'	2	1
191	'142125B '	'M2'	0	1
192	'142125B '	'M3'	0	0
193	'142125C '	' '	1	1
194	'1427968A '	'M1'	1	1
195	'1427968A '	'M2'	1	1
196	'1427968A '	'M3'	0	1

197	'1427968A '	'M1'	1	1
198	'1427968A '	'M2'	1	1
199	'1427968A '	'M3'	0	1
200	'1427968B '	' '	1	1
201	'1429534A '	'M1'	0	0
202	'1429534A '	'M2'	0	0
203	'1429534A '	'M3'	0	0
204	'1429534B '	'M1'	0	0
205	'1429534B '	'M2'	0	0
206	'1429534B '	'M3'	0	0
207	'1429534C '	'M1'	0	0
208	'1429534C '	'M2'	0	0
209	'1429534C '	'M3'	0	0
210	'1430154A '	'M1'	1	1
211	'1430154A '	'M2'	1	1
212	'1430154A '	'M3'	1	1
213	'1430521A '	'M1'	0	0
214	'1430521A '	'M2'	1	1
215	'1430521A '	'M3'	2	0
216	'1430521B '	'M4'	1	1
217	'151429A '	'M1'	1	1
218	'151429A '	'M2'	1	1
219	'151429A '	'M3'	1	1
220	'151429B '	'M1'	1	1
221	'151429B '	'M2'	1	1
222	'151429B '	'M3'	0	0
223	'168107A '	'M1'	2	1
224	'168107A '	'M2'	1	1
225	'168107A '	'M3'	1	1
226	'172322A '	'M1'	1	1
227	'172322A '	'M2'	1	1
228	'172322A '	'M3'	1	1
229	'17255A '	'M1'	2	1
230	'17255A '	'M2'	2	1
231	'17255A '	'M3'	2	1
232	'17255B '	'M1'	2	1
233	'17255B '	'M2'	2	1
234	'17255B '	'M3'	0	0
235	'17255C '	'M1'	0	0
236	'17255C '	'M2'	2	1
237	'17255C '	'M3'	0	0
238	'176180A '	'M1'	1	1
239	'176180A '	'M2'	1	1
240	'176180A '	'M3'	1	1
241	'176180B '	M3''	0	1
242	'178786A '	'M1'	2	1
243	'178786A '	'M2'	1	1
244	'178786A '	'M3'	2	1
245	'1005334A '	'M1'	1	1
246	'1005334A '	'M2'	1	1
247	'1005334A '	'M3'	2	1
248	'1005731A '	'M1'	2	1
249	'1005731A '	'M2'	0	1
250	'1005731A '	'M3'	2	0
251	'1005731B '	'M1'	1	1
252	'1005731B '	'M2'	0	0
253	'1005731B '	'M3'	0	1
254	'1005731C '	'M1'	1	1
255	'1005731C '	'M2'	1	1
256	'1005731C '	'M3'	0	0
257	'1024840A '	'M1'	1	1
258	'1024840A '	'M2'	0	1
259	'1024840A '	'M3'	0	0
260	'1024840B '	'M1'	1	0
261	'1024840B '	'M2'	1	1
262	'1024840B '	'M3'	0	0
263	'1031312A '	'M1'	1	1
264	'1031312A '	'M2'	1	1
265	'1031312A '	'M3'	1	1
266	'1031312A '	'M1'	1	1

267	'1031312A '	'M2'	1	1
268	'1031312A '	'M3'	1	1
269	'1032841A '	'M1'	1	1
270	'1032841A '	'M2'	1	1
271	'1032841A '	'M3'	1	1
272	'1059253A '	'M1'	1	1
273	'1059253A '	'M2'	2	1
274	'1059253A '	'M3'	1	1
275	'1067621A '	'M1'	1	1
276	'1067621A '	'M2'	1	1
277	'1067621A '	'M3'	1	1
278	'1067621B '	'M3'	1	1
279	'106875A '	'M1'	0	1
280	'106875A '	'M2'	1	1
281	'106875A '	'M3'	2	0
282	'1068832A '	'M1'	1	1
283	'1068832A '	'M2'	1	1
284	'1068832A '	'M3'	0	0
285	'1073094A '	'M1'	0	0
286	'1073094A '	'M2'	1	1
287	'1073094A '	'M3'	0	0
288	'1073094B '	'M3'	2	0
289	'107330A '	'M1'	0	0
290	'107330A '	'M2'	0	0
291	'107330A '	'M3'	0	0
292	'107799A '	'M1'	1	1
293	'107799A '	'M2'	1	1
294	'107799A '	'M3'	1	0
295	'1109639A '	'M1'	1	1
296	'1109639A '	'M2'	1	1
297	'1109639A '	'M3'	1	1
298	'1117390A '	'M1'	1	1
299	'1117390A '	'M2'	1	1
300	'1117390A '	'M3'	1	1
301	'1126349A '	'M1'	2	0
302	'1126349A '	'M2'	2	0
303	'1126349A '	'M3'	0	0
304	'1126349B '	'M1'	1	1
305	'1126349B '	'M2'	0	0
306	'1126349B '	'M3'	0	0
307	'1126349C '	'M1'	0	0
308	'1126349C '	'M2'	0	0
309	'1126349C '	'M3'	0	0
310	'1141774A '	'M1'	0	0
311	'1141774A '	'M2'	1	1
312	'1141774A '	'M3'	1	1
313	'115410A '	'M1'	1	1
314	'115410A '	'M2'	1	1
315	'115410A '	'M3'	1	1
316	'115410B '	'M1'	1	1
317	'115410B '	'M2'	1	1
318	'115410B '	'M3'	0	0
319	'115735A '	'M1'	1	1
320	'115735A '	'M2'	1	1
321	'115735A '	'M3'	1	1
322	'1168656A '	'M1'	1	1
323	'1168656A '	'M2'	1	1
324	'1168656A '	'M3'	1	1
325	'1172988A '	'M1'	1	1
326	'1172988A '	'M2'	1	1
327	'1172988A '	'M3'	1	1
328	'1203353A '	'M1'	1	0
329	'1203353A '	'M2'	0	0
330	'1203353A '	'M3'	1	0
331	'1205611A '	'M1'	1	1
332	'1205611A '	'M2'	2	1
333	'1205611A '	'M3'	2	1
334	'1205611B '	'M1'	0	0
335	'1205611B '	'M2'	1	0
336	'1205611B '	'M3'	2	1

337	'1205611C '	'M1'	1	1
338	'1205611C '	'M2'	2	1
339	'1205611C '	'M3'	0	0
340	'1208133A '	'M1'	1	1
341	'1208133A '	'M2'	1	1
342	'1208133A '	'M3'	2	1
343	'1219205A '	'M1'	1	1
344	'1219205A '	'M2'	1	1
345	'1219205A '	'M3'	1	1
346	'1224483A '	'M1'	0	0
347	'1224483A '	'M2'	0	0
348	'1224483A '	'M3'	0	0
349	'1224483B '	'M1'	0	0
350	'1224483B '	'M2'	0	0
351	'1224483B '	'M3'	0	0
352	'122639A '	'M1'	1	1
353	'122639A '	'M2'	1	1
354	'122639A '	'M3'	1	1
355	'1232174A '	'M1'	0	0
356	'1232174A '	'M2'	1	0
357	'1232174A '	'M3'	0	0
358	'1232174B '	'M1'	1	1
359	'1232174B '	'M2'	0	0
360	'1232174B '	'M3'	1	0
361	'1249270A '	'M1'	1	1
362	'1249270A '	'M2'	2	0
363	'1249270A '	'M3'	1	1
364	'1264412A '	'M1'	0	0
365	'1264412A '	'M2'	2	0
366	'1264412A '	'M3'	0	0
367	'1275560A '	'M1'	0	0
368	'1275560A '	'M2'	1	1
369	'1275560A '	'M3'	0	0
370	'1275560B '	'M1'	1	1
371	'1275560B '	'M2'	1	1
372	'1275560B '	'M3'	1	1
373	'1275560C '	'M1'	1	1
374	'1275560C '	'M2'	1	1
375	'1275560C '	'M3'	0	0
376	'1283956A '	'M1'	1	1
377	'1283956A '	'M2'	2	0
378	'1283956A '	'M3'	0	0
379	'1283956B '	'M3'	1	1
380	'1283974A '	'M1'	0	0
381	'1283974A '	'M2'	0	0
382	'1283974A '	'M3'	0	0
383	'1283974B '	'M1'	0	0
384	'1283974B '	'M2'	0	0
385	'1283974B '	'M3'	0	0
386	'1283974C '	'M3'	0	1
387	'1284970A '	'M1'	1	1
388	'1284970A '	'M2'	1	1
389	'1284970A '	'M3'	2	1
390	'1284970B '	'M3'	1	1
391	'1308000A '	'M1'	2	0
392	'1308000A '	'M2'	0	0
393	'1308000A '	'M3'	0	0
394	'1308000B '	'M1'	0	0
395	'1308000B '	'M2'	0	0
396	'1308000B '	'M3'	2	0
397	'1308000C '	'M1'	0	0
398	'1308000C '	'M2'	0	0
399	'1308000C '	'M3'	0	0
400	'1312854A '	'M1'	1	1
401	'1312854A '	'M2'	0	0
402	'1312854A '	'M3'	1	1
403	'1312854B '	'M1'	1	1
404	'1312854B '	'M2'	1	1
405	'1312854B '	'M3'	0	0
406	'1312854C '	'M1'	0	0

407	'1312854C '	'M2'	2	1
408	'1312854C '	'M3'	0	0
409	'1315254A '	'M1'	1	1
410	'1315254A '	'M2'	1	1
411	'1315254A '	'M3'	0	0
412	'1315254B '	'M3'	1	1
413	'1334091A '	'M1'	1	1
414	'1334091A '	'M2'	1	1
415	'1334091A '	'M3'	1	1
416	'1350710A '	'M1'	1	1
417	'1350710A '	'M2'	2	0
418	'1350710A '	'M3'	0	0
419	'1350710B '	'M1'	1	1
420	'1350710B '	'M2'	1	1
421	'1350710B '	'M3'	0	0
422	'1350710C '	'M1'	1	1
423	'1350710C '	'M2'	0	0
424	'1350710C '	'M3'	0	0
425	'1380703A '	'M1'	0	0
426	'1380703A '	'M2'	0	0
427	'1380703A '	'M3'	0	0
428	'1380703B '	'M1'	1	1
429	'1380703B '	'M2'	2	1
430	'1380703B '	'M3'	1	1
431	'1380703C '	'M3'	1	1
432	'138365A '	'M1'	1	1
433	'138365A '	'M2'	2	0
434	'138365A '	'M3'	2	0
435	'138365B '	'M1'	0	0
436	'138365B '	'M2'	0	0
437	'138365B '	'M3'	1	1
438	'1385356A '	'M1'	1	1
439	'1385356A '	'M2'	1	1
440	'1385356A '	'M3'	1	1
441	'1386301A '	'M1'	1	1
442	'1386301A '	'M2'	1	1
443	'1386301A '	'M3'	1	1
444	'1405573A '	'M1'	1	1
445	'1405573A '	'M2'	1	1
446	'1405573A '	'M3'	1	1
447	'1405573B '	'M3'	1	1
448	'1417502A '	'M1'	1	1
449	'1417502A '	'M2'	1	1
450	'1417502A '	'M3'	0	0
451	'1417502B '	'M1'	1	1
452	'1417502B '	'M2'	1	1
453	'1417502B '	'M3'	0	0
454	'1421129A '	'M1'	1	1
455	'1421129A '	'M2'	1	1
456	'1421129A '	'M3'	1	1
457	'1421129B '	'M1'	2	1
458	'1421129B '	'M2'	1	1
459	'1421129B '	'M3'	0	0
460	'1439152A '	'M1'	1	1
461	'1439152A '	'M2'	2	0
462	'1439152A '	'M3'	1	1
463	'1439152B '	'M3'	1	1
464	'1440192A '	'M1'	0	0
465	'1440192A '	'M2'	2	0
466	'1440192A '	'M3'	0	0
467	'1440192B '	'M1'	0	0
468	'1440192B '	'M2'	1	1
469	'1440192B '	'M3'	1	1
470	'146585A '	'M1'	0	0
471	'146585A '	'M2'	1	1
472	'146585A '	'M3'	1	1
473	'146585B '	'M3'	1	1
474	'165826A '	'M1'	1	1
475	'165826A '	'M2'	1	1
476	'165826A '	'M3'	1	1

477	'207038A	'	'M1'	0	0
478	'207038A	'	'M2'	0	0
479	'207038A	'	'M3'	0	0
480	'207038B	'	'M1'	1	1
481	'207038B	'	'M2'	0	1
482	'207038B	'	'M3'	2	1
483	'207038C	'	'M1'	2	1
484	'207038C	'	'M2'	0	0
485	'207038C	'	'M3'	0	0
486	'208679A	'	'M1'	1	0
487	'208679A	'	'M2'	0	0
488	'208679A	'	'M3'	0	0
489	'208679B	'	'M1'	0	0
490	'208679B	'	'M2'	0	0
491	'208679B	'	'M3'	0	0
492	'208679C	'	'M1'	1	1
493	'208679C	'	'M2'	0	0
494	'208679C	'	'M3'	0	0
495	'214353A	'	'M1'	1	1
496	'214353A	'	'M2'	1	1
497	'214353A	'	'M3'	0	0
498	'214353B	'	'M1'	1	1
499	'214353B	'	'M2'	1	1
500	'214353B	'	'M3'	0	0
501	'234191A	'	'M1'	1	1
502	'234191A	'	'M2'	1	1
503	'234191A	'	'M3'	1	1
504	'234191B	'	'M1'	2	1
505	'234191B	'	'M2'	1	1
506	'234191B	'	'M3'	1	1
507	'234191C	'	'M3'	2	0
508	'236898A	'	'M1'	1	1
509	'236898A	'	'M2'	1	1
510	'236898A	'	'M3'	1	1
511	'236898B	'	'M1'	1	1
512	'236898B	'	'M2'	1	1
513	'236898B	'	'M3'	0	0
514	'250913A	'	'M1'	2	1
515	'250913A	'	'M2'	0	0
516	'250913A	'	'M3'	0	1
517	'264834A	'	'M1'	2	1
518	'264834A	'	'M2'	0	0
519	'264834A	'	'M3'	0	0
520		''	'M1'	1	1
521		''	'M2'	1	1
522		''	'M3'	0	0
523	'267221B	'	'M3'	1	1
524	'275797A	'	'M1'	0	0
525	'275797A	'	'M2'	0	0
526	'275797A	'	'M3'	2	1
527	'29101A	'	'M1'	1	1
528	'29101A	'	'M2'	1	1
529	'29101A	'	'M3'	1	1
530	'29101B	'	'M3'	1	1
531	'295152A	'	'M1'	0	0
532	'295152A	'	'M2'	0	0
533	'295152A	'	'M3'	1	1
534	'295608A	'	'M1'	2	1
535	'295608A	'	'M2'	1	1
536	'295608A	'	'M3'	1	1
537	'303192A	'	'M1'	0	0
538	'303192A	'	'M2'	1	1
539	'303192A	'	'M3'	1	1
540	'303192B	'	'M1'	0	0
541	'303192B	'	'M2'	2	0
542	'303192B	'	'M3'	0	0
543	'334232A	'	'M1'	0	1
544	'334232A	'	'M2'	1	1
545	'334232A	'	'M3'	0	0
546	'334232B	'	'M1'	2	0

547	'334232B	'	'M2'	0	0
548	'334232B	'	'M3'	0	0
549	'335727A	'	'M1'	1	1
550	'335727A	'	'M2'	0	0
551	'335727A	'	'M3'	0	0
552	'335727B	'	'M1'	1	1
553	'335727B	'	'M2'	0	0
554	'335727B	'	'M3'	0	0
555	'335727C	'	'M1'	1	1
556	'335727C	'	'M2'	1	1
557	'335727C	'	'M3'	0	0
558	'349141A	'	'M1'	0	0
559	'349141A	'	'M2'	0	0
560	'349141A	'	'M3'	1	1
561	'349141B	'	'M1'	0	1
562	'349141B	'	'M2'	0	0
563	'349141B	'	'M3'	0	0
564	'349141C	'	'M1'	0	0
565	'349141C	'	'M2'	0	0
566	'349141C	'	'M3'	0	0
567	'352806A	'	'M1'	1	1
568	'352806A	'	'M2'	1	1
569	'352806A	'	'M3'	1	1
570	'352806B	'	'M3'	1	1
571	'391738A	'	'M1'	1	1
572	'391738A	'	'M2'	1	1
573	'391738A	'	'M3'	0	0
574	'391738B	'	'M1'	1	1
575	'391738B	'	'M2'	0	0
576	'391738B	'	'M3'	0	0
577	'391738C	'	'M1'	0	0
578	'391738C	'	'M2'	0	0
579	'391738C	'	'M3'	0	0
580	'395224A	'	'M1'	1	1
581	'395224A	'	'M2'	1	1
582	'395224A	'	'M3'	1	1
583	'395224B	'	'M1'	1	1
584	'395224B	'	'M2'	1	1
585	'395224B	'	'M3'	2	0
586	'422138A	'	'M1'	2	0
587	'422138A	'	'M2'	0	0
588	'422138A	'	'M3'	0	0
589	'422138B	'	'M1'	1	1
590	'422138B	'	'M2'	2	0
591	'422138B	'	'M3'	1	1
592	'42413A	'	'M1'	0	0
593	'42413A	'	'M2'	2	0
594	'42413A	'	'M3'	0	0
595	'42413C	'	'M3'	0	0
596	'43091A	'	'M1'	1	1
597	'43091A	'	'M2'	2	1
598	'43091A	'	'M3'	2	1
599	'432717A	'	'M1'	1	1
600	'432717A	'	'M2'	1	1
601	'432717A	'	'M3'	1	1
602	'437244A	'	'M1'	1	1
603	'437244A	'	'M2'	1	1
604	'437244A	'	'M3'	1	1
605	'437795A	'	'M1'	1	1
606	'437795A	'	'M2'	1	1
607	'437795A	'	'M3'	1	1
608	'461451A	'	'M1'	1	1
609	'461451A	'	'M2'	1	1
610	'461451A	'	'M3'	1	1
611	'461451B	'	'M3'	1	1
612	'486834A	'	'M1'	1	1
613	'486834A	'	'M2'	1	1
614	'486834A	'	'M3'	1	1
615	'505253A	'	'M1'	2	0
616	'505253A	'	'M2'	0	1

617	'505253A	'	'M3'	2	1
618	'521582A	'	'M1'	1	1
619	'521582A	'	'M2'	1	1
620	'521582A	'	'M3'	1	1
621	'521582B	'	'M1'	1	1
622	'521582B	'	'M2'	1	1
623	'521582B	'	'M3'	1	1
624	'521582C	'	'M1'	1	1
625	'521582C	'	'M2'	1	1
626	'521582C	'	'M3'	0	0
627	'534076A	'	'M1'	1	1
628	'534076A	'	'M2'	1	1
629	'534076A	'	'M3'	1	1
630	'539867A	'	'M1'	1	1
631	'539867A	'	'M2'	1	1
632	'539867A	'	'M3'	1	1
633	'539867B	'	'M1'	1	1
634	'539867B	'	'M2'	1	1
635	'539867B	'	'M3'	1	1
636	'539867D	'	'M1'	1	1
637	'539867D	'	'M2'	1	1
638	'539867D	'	'M3'	0	0
639	'539949A	'	'M1'	0	0
640	'539949A	'	'M2'	0	0
641	'539949A	'	'M3'	0	0
642	'539949B	'	'M1'	0	0
643	'539949B	'	'M2'	0	0
644	'539949B	'	'M3'	0	0
645	'560305A	'	'M1'	0	0
646	'560305A	'	'M2'	1	1
647	'560305A	'	'M3'	1	1
648	'568293A	'	'M1'	1	1
649	'568293A	'	'M2'	1	1
650	'568293A	'	'M3'	2	0
651	'568293B	'	'M3'	2	0
652	'596656A	'	'M1'	2	0
653	'596656A	'	'M2'	0	0
654	'596656A	'	'M3'	1	1
655	'596656B	'	'M1'	1	1
656	'596656B	'	'M2'	1	1
657	'596656B	'	'M3'	1	1
658		''	'M1'	0	0
659		''	'M2'	0	0
660		''	'M3'	0	0
661	'613063B	'	'M1'	0	0
662	'613063B	'	'M2'	0	0
663	'613063B	'	'M3'	0	0
664	'613063C	'	'M1'	1	1
665	'613063C	'	'M2'	0	0
666	'613063C	'	'M3'	0	0
667	'620081A	'	'M1'	1	1
668	'620081A	'	'M2'	1	1
669	'620081A	'	'M3'	0	0
670	'620081B	'	'M3'	1	1
671	'62284A	'	'M1'	1	1
672	'62284A	'	'M2'	1	1
673	'62284A	'	'M3'	1	1
674	'62284B	'	'M1'	1	1
675	'62284B	'	'M2'	0	0
676	'62284B	'	'M3'	0	0
677	'62284C	'	'M1'	0	0
678	'62284C	'	'M2'	0	0
679	'62284C	'	'M3'	0	0
680	'646336A	'	'M1'	1	1
681	'646336A	'	'M2'	1	1
682	'646336A	'	'M3'	0	0
683	'646336B	'	'M1'	1	1
684	'646336B	'	'M2'	1	1
685	'646336B	'	'M3'	1	1
686	'659523A	'	'M1'	1	1

687	'659523A	'	'M2'	1	1
688	'659523A	'	'M3'	1	0
489	'659523B	'	'M3'	1	1
690	'679695A	'	'M1'	1	1
691	'679695A	'	'M2'	1	1
692	'679695A	'	'M3'	1	1
693	'81128A	'	'M1'	2	1
694	'81128A	'	'M2'	1	1
695	'81128A	'	'M3'	0	0
696	'81128B	'	'M3'	1	1
697	'815592A	'	'M1'	1	1
698	'815592A	'	'M2'	0	1
699	'815592A	'	'M3'	2	0
700	'815592B	'	'M1'	1	1
601	'815592B	'	'M2'	1	1
702	'815592B	'	'M3'	1	1
703	'815592C	'	'M1'	1	1
704	'815592C	'	'M2'	1	1
705	'815592C	'	'M3'	0	0
706	'82614A	'	'M1'	0	1
707	'82614A	'	'M2'	2	1
708	'82614A	'	'M3'	1	1
709	'82614B	'	'M3'	1	1
710	'846208A	'	'M1'	1	1
711	'846208A	'	'M2'	0	0
712	'846208A	'	'M3'	0	0
713	'846208B	'	'M3'	1	1
714	'8778A	'	'M1'	2	1
715	'8778A	'	'M2'	0	1
716	'8778A	'	'M3'	0	1
717	'88918A	'	'M1'	1	1
718	'88918A	'	'M2'	1	1
719	'88918A	'	'M3'	1	1
720	'88918B	'	'M1'	1	1
721	'88918B	'	'M2'	1	1
722	'88918B	'	'M3'	1	1
723	'88918C	'	'M3'	0	1
724	'889587A	'	'M1'	1	1
725	'889587A	'	'M2'	1	1
726	'889587A	'	'M3'	1	1
727	'891147A	'	'M1'	0	0
728	'891147A	'	'M2'	0	0
729	'891147A	'	'M3'	1	1
730	'896205A	'	'M1'	1	1
731	'896205A	'	'M2'	1	1
732	'896205A	'	'M3'	1	1
733	'913224A	'	'M1'	1	1
734	'913224A	'	'M2'	1	1
735	'913224A	'	'M3'	1	1
736	'913224B	'	'M3'	1	1
737	'92001AA	'	'M1'	0	0
738	'92001AA	'	'M2'	1	1
739	'92001AA	'	'M3'	1	1
740	'922545A	'	'M1'	1	1
741	'922545A	'	'M2'	1	1
742	'922545A	'	'M3'	1	1
743	'93428A	'	'M1'	1	1
744	'93428A	'	'M2'	1	1
745	'93428A	'	'M3'	1	1
746	'94261A	'	'M1'	1	1
747	'94261A	'	'M2'	1	1
748	'94261A	'	'M3'	1	1
749	'94261B	'	'M3'	1	1
750	'951186A	'	'M1'	1	1
751	'951186A	'	'M2'	1	1
752	'951186A	'	'M3'	1	1
753	'95556A	'	'M1'	2	0
754	'95556A	'	'M2'	1	1
755	'95556A	'	'M3'	2	1
756	'95556B	'	'M3'	1	1

757	'968486A	'	'M1'	1	1
758	'968486A	'	'M2'	1	1
759	'968486A	'	'M3'	1	1
760	'968486B	'	'M1'	0	1
761	'968486B	'	'M2'	1	1
762	'968486B	'	'M3'	0	1
763	'968486C	'	'M1'	1	1
764	'968486C	'	'M2'	0	0
765	'968486C	'	'M3'	0	0
766	'973856AA	'	'M1'	1	1
767	'973856AA	'	'M2'	1	1
768	'973856AA	'	'M3'	1	1
769	'973856AB	'	'M3'	1	1
770	'9756A	'	'M1'	0	0
771	'9756A	'	'M2'	1	1
772	'9756A	'	'M3'	1	1
773	'9756B	'	'M3'	1	1
774	'9756B	'	'PRE'	1	1
775	'9756B	'	'POST'	1	1
776	'999563A	'	'M1'	1	1
777	'999563A	'	'M2'	2	1
778	'999563A	'	'M3'	2	1
